# Congenital, Intrapartum and Postnatal Maternal-Fetal-Neonatal SARS-CoV-2 Infections: A Narrative Review

**DOI:** 10.3390/nu12113570

**Published:** 2020-11-20

**Authors:** Rafael A. Caparros-Gonzalez, María Angeles Pérez-Morente, Cesar Hueso-Montoro, María Adelaida Álvarez-Serrano, Alejandro de la Torre-Luque

**Affiliations:** 1 Department of Nursing, Faculty of Health Sciences, University of Granada, 18006 Granada, Spain; rcg477@ugr.es (R.A.C.-G.); cesarhueso@ugr.es (C.H.-M.); 2 Mind, Brain and Behavior Research Center (CIMCYC), Faculty of Psychology, University of Granada, 18011 Granada, Spain; 3 Department of Nursing, Faculty of Health Sciences, University of Jaen, 23071 Jaen, Spain; 4 Department of Nursing, Faculty of Health Sciences, University of Granada, 51001 Ceuta, Spain; adealvarez@ugr.es; 5 Department of Legal Medicine, Psychiatry and Pathology, Faculty of Medicine, Universidad Complutense de Madrid, 28040 Madrid, Spain; af.delatorre@ucm.es; 6 Center for Biomedical Research in Mental Health (CIBERSAM), 28029 Madrid, Spain

**Keywords:** SARS virus, COVID-19, pregnancy, transmission, neonates, breastmilk

## Abstract

Background: There is inconclusive evidence regarding congenital, intrapartum, and postnatal maternal-fetal-neonatal SARS-CoV-2 infections during the COVID-19 pandemic. A narrative review was conducted with the aim of guiding clinicians on the management of pregnant women with respect to congenital, intrapartum, and postnatal maternal-fetal-neonatal SARS-CoV-2 infections and breastfeeding during the COVID-19 pandemic. Methods: Searches were conducted in Web of Science, PubMed, Scopus, Dialnet, CUIDEN, Scielo, and Virtual Health Library to identify observational, case series, case reports, and randomized controlled trial studies assessing the transmission of SARS-CoV-2 from mother to baby and/or through breastfeeding during the COVID-19 pandemic. Results: A total of 49 studies was included in this review, comprising 329 pregnant women and 331 neonates (two pregnant women delivered twins). The studies were performed in China (*n* = 26), USA (*n* = 7), Italy (*n* = 3), Iran (*n* = 2), Switzerland (*n* = 1), Spain (*n* = 1), Turkey (*n* = 1), Australia (*n* = 1), India (*n* = 1), Germany (*n* = 1), France (*n* = 1), Canada (*n* = 1), Honduras (*n* = 1), Brazil (*n* = 1), and Peru (*n* = 1). Samples from amniotic fluid, umbilical cord blood, placenta, cervical secretion, and breastmilk were collected and analyzed. A total of 15 placental swabs gave positive results for SARS-CoV-2 ribonucleic acid (RNA) on the fetal side of the placenta. SARS-CoV-2 RNA was found in seven breastmilk samples. One umbilical cord sample was positive for SARS-CoV-2. One amniotic fluid sample tested positive for SARS-CoV-2. Conclusions: This study presents some evidence to support the potential of congenital, intrapartum, and postnatal maternal-fetal-neonatal SARS-CoV-2 infections during the COVID-19 pandemic. Mothers should follow recommendations including wearing a facemask and hand washing before and after breastfeeding.

## 1. Introduction

The new coronavirus disease (COVID-19) has rapidly spread around the world since its first identification in Wuhan (China) [1]. COVID-19 is caused by a coronavirus named SARS-CoV-2 which is associated with previously detected diseases such as Severe Acute Respiratory Syndrome (SARS), Middle East Respiratory Syndrome (MERS-CoV), and the common cold. The World Health Organization (WHO) characterized the COVID-19 outbreak as a pandemic due to its rapid and massive spread [2].

In this respect, pregnancy represents a vulnerable period, and this pandemic can negatively impact its outcomes [1,3]. Pregnant women may, therefore, be a group requiring special care in relation to the transmission of SARS-CoV-2 [1]. It has been reported that testing for SARS-CoV-2 in breastmilk, amniotic fluid, or cord blood has given negative results [4,5,6].

Nevertheless, limited data are available on the impact that COVID-19 may have during pregnancy [1,7]. Transmission from mother-to-child has not been confirmed. However, breastfeeding is sometimes not recommended [8]. The aim of this study was to clarify whether the transmission of SARS-CoV-2 could occur in utero (congenital), intrapartum, and/or postnatally through breastmilk, amniotic fluid, cord blood, cervical secretion, or the placenta and to provide updated evidence on breastfeeding during the COVID-19 pandemic.

## 2. Materials and Methods

A narrative review was conducted between 25 August 2020 and 15 September 2020. The search was updated on 10 November 2020. Search queries to select studies were performed using Medical Subject Headings (MeSH) (“pregnant*”, “COVID-19”, “SARS-CoV-2”, “Breast Feeding”).

The inclusion criteria were observational studies, case series, reports, and randomized controlled trial studies assessing the transmission of SARS-CoV-2 from mother to baby, breastfeeding in times of the COVID-19 pandemic, or both. Due to the novelty of the topic, some results might have been published as letters to the editor. For this reason, letters were selected. No language or date of publication restrictions was applied. Systematic reviews, rapid reviews, scoping reviews, meta-analyses, and animal articles looking at breastmilk, amniotic fluid, cord blood, cervical secretion, or the placenta were excluded.

**Figure 1:**
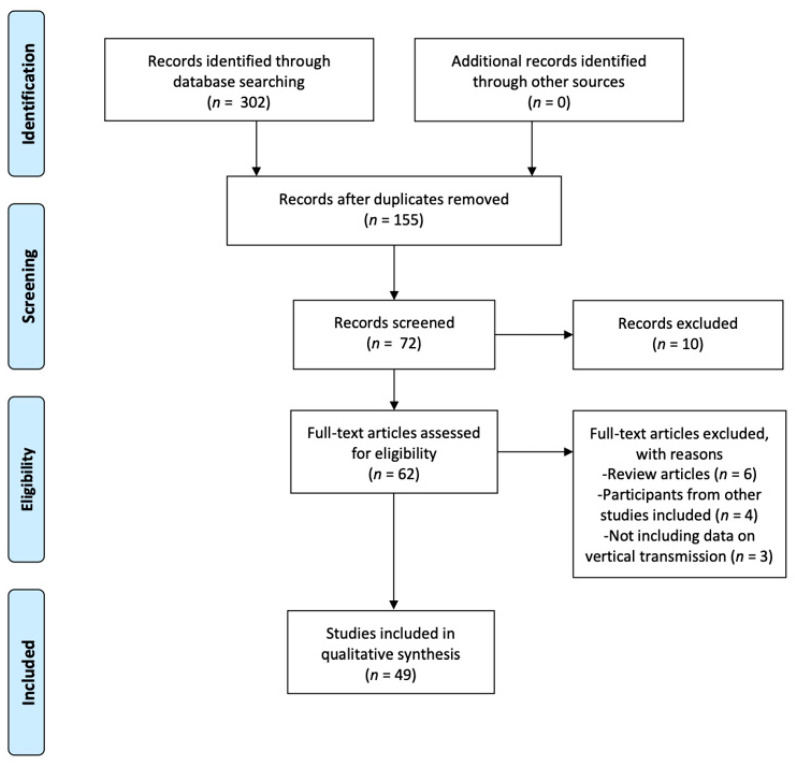
Flow diagram of our search for and selection of studies. Adapted from Preferred Reporting Items for Systematic Reviews and Meta-Analyses (PRISMA statement).

The databases in which the search was conducted included Web of Science (WOS), PubMed, Scopus, Dialnet, CUIDEN, Scielo, and Virtual Health Library (VHL). Figure 1 provides information on the Preferred Reporting Items for Systematic Reviews and Meta-Analyses (PRISMA) flow diagram [9], which offers an overview of the search, selection, and inclusion process for the articles included in this review.

Due to this study being a review, no ethical approval was required. This review was registered in PROSPERO with the number CRD42020182325.

## 3. Results

A total of 302 studies was selected. The search on WOS provided 25 hits. The search on PubMed offered 162 outcomes, while 86 studies were found through SCOPUS. Twenty-nine results were identified through VHL. No studies were found on Dialnet, CUIDEN, or Scielo. See Figure 1 for a detailed view of the selection and inclusion process.

Forty-nine studies were included in this review after assessing their eligibility (10–58). All the studies (*n* = 49) selected in this review were case studies or case reports published in 2020. A pool of 329 pregnant women and their 331 neonates (two pregnant women delivered twins) was derived from the selected studies. Nevertheless, an overlap in cases might be present. The majority of studies were performed in China (*n* = 26), and the rest were performed in the USA (*n* = 7), Italy (*n* = 3), Iran (*n* = 2), Switzerland (*n* = 1), Spain (*n* = 1), Turkey (*n* = 1), Australia (*n* = 1), India (*n* = 1), Germany (*n* = 1), France (*n* = 1), Canada (*n* = 1), Honduras (*n* = 1), Brazil (*n* = 1), and Peru (*n* = 1).

### 3.1. Congenital, Intrapartum, and Postnatal Maternal-Fetal-Neonatal SARS-CoV-2 Infection

Maternal-fetal-neonatal SARS-CoV-2 infection can occur in utero (following maternal viremia and placental infection), intrapartum (via cervical or vaginal secretions), or after birth via breast milk. The detection of the virus by **p**olymerase **c**hain **r**eaction (PCR) in umbilical cord blood or neonatal blood collected within the first 12 h of birth or amniotic fluid collected prior to the rupture of the membrane gives evidence for a congenital infection [10]. A narrative synthesis is presented below.

#### 3.1.1. Amniotic Fluid

Amniotic fluid was assessed from pregnant women with a positive result for COVID-19 in 11 studies [11,12,13,14,15,16,17,18,19,20,21]. Only one study reported a positive result for SARS-CoV-2 [20]. This study was a case report conducted in Iran on a 22-year-old woman who delivered a baby at 33 weeks of gestation through a C-section. Throat swab samples were obtained and analyzed from the neonate over several days. This neonate had a positive result for SARS-CoV-2 24 h after birth and a week later. The neonate had no severe symptoms. The mother in this study died due to respiratory complications [22]. No SARS-CoV-2 infection was found in the rest of studies that evaluated amniotic fluid [11,12,13,14,15,16,17,18,19,20,21].

#### 3.1.2. Umbilical Cord Blood

A total of 12 studies looked for SARS-CoV-2 infection in umbilical cord blood [11,12,13,14,15,16,18,20,23,24,25,26]. In 10 studies, umbilical cord blood samples tested negative for SARS-CoV-2 [11,12,13,14,18,22,23,27,28,29]. Two studies reported a positive result for SARS-CoV-2 RNA in umbilical cord samples [15,26]. No blood sample was collected or assessed from the umbilical cord in one of these studies [26]. Instead, the positive result obtained for SARS-CoV-2 was reported to be from the umbilical cord stump [26]. According to the authors, the umbilical cord stump was collected observing strict aseptic precautions and following the Indian national guidelines at birth to minimize the contamination of samples. Both neonates in these studies had a negative result for SARS-CoV-2 [15,26].

#### 3.1.3. Placenta

Placental tissue was analyzed in 17 studies [12,14,15,16,17,21,25,26,29,30,31,32,33,34,35,36,37]. Although the placentas tested negative in six studies [12,14,16,17,30,31], a total of 16 placental swabs tested positive for SARS-CoV-2 RNA on the fetal side of the placenta [15,21,25,26,29,32,33,34,35,36,37]. SARS-CoV-2 RNA has been found to be present in the syncytiotrophoblast [34,36,37,38], cytotrophoblast [34], chorionic villi endothelial cells, and in trophoblasts [35] among samples from pregnant women who tested positive for SARS-CoV-2. COVID-19 coronavirus RNA was found in the placenta of a PCR-negative pregnant woman. This woman had SARS-CoV-2 antibodies 10 days after birth [26]. Besides, five placental showed areas of inflammatory infiltrate and infarction [21,25,33,36,37]. Among those studies reporting a positive result for SARS-CoV-2 RNA in the placenta [15,21,25,26,29,32,33,34,35,36,37], six neonates resulted positive for SARS-CoV-2 [21,26,29,33,36].

#### 3.1.4. Cervical Secretion

Maternal cervical secretion was only assessed in three case report studies [14,17,21]. One study assessing maternal cervical secretion reported a positive test for SARS-CoV-2 [21]. The neonate in this study had a positive test for SARS-CoV-2 [21].

#### 3.1.5. Breast Milk

SARS-CoV-2 RNA has been found in seven studies [33,39,40,41,42,43,44]. Using Real-Time Quantitative Reverse Transcription-PCR (qRT-PCR) for SARS-CoV-2 *N* and *ORF1b-nsp14* genes for SARS-CoV-2 detection, SARS-CoV-2 RNA was found in human breast milk from both the right and left breast between 10–15 days after birth [39,40]. SARS-CoV-2 RNA was detected in colostrum and breast milk by SARS-CoV-2 RT-PCR testing at 8, 72, and 96 h after birth [41] and even after storing a sample of breast milk at −80 °C for 30 days [42]. In this study, a preterm newborn was fed using SARS-CoV-2-positive breast milk. The preterm newborn nasopharyngeal swabs were negative for SARS-CoV-2 RNA [42]. An additional study reported a positive case for SARS-CoV-2 RNA in breast milk, but information on sample collection was not provided [33]. Two further studies found a positive result for SARS-CoV-2 Immunoglobulin G (IgG), but breast milk was negative for SARS-CoV-2 Immunoglobulin M (IgM) and SARS-CoV-2 RNA in the same sample [45,46].

SARS-CoV-2 was not found in six studies analyzing breast milk samples [11,12,13,14,17,27].

In total, 321 neonates were fed with formula. Ten neonates in this review were breastfed while their mothers were wearing a mask [29,33,35,39,40,41,42,43,44,45]. Although those mothers wore a mask to breastfeed their neonates, five neonates had a positive result for SARS-CoV-2 [33,39,40,41,42].

The main results of the included studies are described in Table 1. Table 1 displays information on the city and country where a certain study was performed, the study design, number of pregnant women participating in each study, maternal age, number of fetuses participating in each study, trimester of pregnancy when the COVID-19 diagnosis was performed, number of pregnant women having a positive test for SARS-CoV-2, gestational age at birth, neonates having a positive throat swab for SARS-CoV-2 (yes = 1; no = 0), potential and confirmed maternal source of neonatal SARS-CoV-2 infection, neonatal feeding method (breast milk versus formula), and number of vaginally born neonates.

## 4. Discussion

There is limited evidence on the detrimental impact that COVID-19 may have on pregnancy [1,7]. Inconsistencies exist regarding congenital, intrapartum, and postnatal maternal-fetal-neonatal SARS-CoV-2 infections [8]. Although the benefits that breast milk can have on neonatal health have been reported [60], women are recommended not to breastfeed their neonates during the COVID-19 pandemic [13,16,20,21,26,27,31,47,49,55,59]. The aim of this study was to gather the most up-to-date evidence on the congenital and intrapartum transmission of SARS-CoV-2 from mother-to-child and to extract integrated conclusions from the existing literature. Potential postnatal transmission through breast milk was also considered.

Studies included in this review were all case reports and case series assessing the congenital, intrapartum, and postnatal maternal-fetal-neonatal transmission of SARS-CoV-2. All pregnant women included in the studies had a positive throat swab test for SARS-CoV-2 infection.

### 4.1. Amniotic Fluid

Studies assessing amniotic fluid comprised of 10 studies. Most of the neonates among these 10 studies were delivered by C-section in a negative-pressure room [11,12,13,14,16,17,18,19,20,21]. The amniotic fluid samples assessed in those studies were collected after the rupture of membranes in an operating room at the time of a C-section in eight studies [11,12,14,16,18,19,20,21]. These studies reported and guaranteed that the amniotic fluid samples were not contaminated and represented fetal intrauterine conditions [11,12,14,16,18,19,20,21]. The bulk of the studies reported a negative test for SARS-CoV-2 in amniotic fluid specimens. This finding is in line with previous studies concluding that the transmission of SARS-CoV-2 through amniotic fluid during the COVID-19 pandemic was unlikely to occur [1,3]. Unfortunately, those studies were based on samples collected during the early stages of the COVID-19 outbreak [1,3]. In the present review, one study reported a positive test for SARS-CoV-2 inflection in amniotic fluid from a pregnant woman [20]. Due to her severe health state, the pregnant woman in this case report had a C-section and a preterm neonate was born at 33 weeks of gestation. This neonate had a positive result for SARS-CoV-2 but with no respiratory distress. The neonate was fed with formula. Due to respiratory complications, the mother died 16 days after the C-section. An absence of viral RNA was determined from amniotic fluid specimens tested by reverse transcriptase-polymerase chain reaction (RT-PCR) among pregnant women who were positive for previous coronavirus diseases (SARS or MERS) [61]. The findings from studies assessing amniotic fluid suggest that the transmission of SARS-CoV-2 through the amniotic fluid is unlikely to occur. Only one study in this review informed of a positive result for SARS-CoV-2 in amniotic fluid [61]. This finding might have been due to the contamination of the amniotic fluid sample.

### 4.2. Umbilical Cord Blood

Ten studies assessing umbilical cord blood samples reported a negative result for SARS-CoV-2, including [11,12,13,14,16,18,20,23,24,25]. Negative results for SARS-CoV-2 infection in cord blood have been previously reported [1,3]. These findings support some studies that found no evidence of the viral shedding of SARS or MERS in umbilical cord blood [61,62,63].

In this review, we included a single study reporting a positive result for SARS-CoV-2 in the umbilical stump from a neonate who tested positive for SARS-CoV-2 [26]. This same study reported a positive result for SARS-CoV-2 in the placenta [26]. Delayed cord clamping was not performed in this case study [26]. The mother’s nasopharyngeal aspirate was negative for SARS-CoV-2 on the day of admission and at day five. However, the mother tested positive for antibodies 10 days after delivery [26]. None of the studies included in this review reported the presence of SARS-CoV-2 in umbilical cord blood [11,12,13,14,16,18,20,23,24,25].

Only one study found SARS-CoV-2 in the umbilical cord; this finding was gathered from the umbilical cord stump but not from umbilical cord blood [26]. This finding suggests that SARS-CoV-2 might not cross the placenta barrier through the umbilical cord. Extreme caution should be maintained when manipulating biological measures. It is recommended that protocols are strictly followed to obtain the most accurate results.

### 4.3. Placenta

A total of 11 studies reported a positive test for SARS-CoV-2 RNA in placental tissues [21,25,26,29,32,33,34,35,36,37,38]. It has been reported that SARS-CoV-2 can bind to the angiotensin-converting enzyme 2 (ACE2) receptors in the placenta for cell entry [64]. The fact that SARS-CoV-2 may cross the placental barrier by means of binding to the ACE2 receptor supports the potential risk of the mother-to-infant transmission of SARS-CoV-2 [38]. A study in this review reported a second-trimester miscarriage in a pregnant woman who was positive for SARS-CoV-2 [25]. The miscarriage in this study appeared to be related to a placental infection with SARS-CoV-2. Nevertheless, fetal samples from the anus, liver, thymus, and lung tested negative for SARS-CoV-2 [25]. On the contrary, a previous study presented some evidence on the unlikelihood of SARS-CoV-2 infecting the placenta, despite ACE2 being minimally expressed in the placenta during pregnancy [65]. Certain viruses such as the Zika virus were found to evade the protection that the placenta barrier confers. Due to the effects that a congenital Zika infection can have, thousands of microcephalic neonates were reported in 2016 [66]. Six studies analyzing the placenta samples in this review gave a negative result for SARS-CoV-2 [12,14,16,17,30,31]. These results are in line with studies on SARS that were unable to find coronavirus in placentas [60,61,62]. Although no SARS was found in some of the placenta specimens, these placentas were reported to present increased subchorionic, intervillous fibrin, thrombotic vasculopathy, and areas of avascular chorionic villi, which are associated with fetal vascular malperfusion and fetal intrauterine growth restriction [62].

The fact that SARS-CoV-2 RNA has been found in placental tissues from 11 studies in this review [21,25,26,29,32,33,34,35,36,37,38] reflects the potential transmission of SARS-CoV-2 from mother to fetus. In fact, seven studies with a positive result for SARS-CoV-2 reported a neonatal positive throat swab for SARS-CoV-2 [21,25,26,29,34,36,37]. Although the placenta may play a key role in protecting fetuses against a SARS-CoV-2 infection [65], this might not be always the case.

### 4.4. Breastfeeding

The United Nations Children’s Fund has asserted that breastfeeding provides neonates with a range of micronutrients that may protect them against infections [67]. The WHO has stated that every neonate in the world should be breastfed for at least 6 months [68]. However, only eight studies included in this review reported that neonates were breastfed [29,33,35,39,40,41,42,45]. Breast milk samples had a positive result for SARS-CoV-2 infection in seven studies that evaluated human milk from mothers infected with the same virus [33,39,40,41,42,43,44] Five studies in this review reported a positive neonatal case for SARS-CoV-2 among mothers with a positive result for SARS-CoV-2 in breast milk [33,39,40,41,42]. It should be clarified whether those neonates were positive for SARS-CoV-2 before or after they were breastfed. Future studies should take into consideration the potential contamination of breast milk from neonates’ saliva while breastfeeding. None of the studies in this review attempted to culture the SARS-CoV-2 from breast milk samples. Although SARS-CoV-2 RNA has been isolated from breast milk samples, it is still unclear whether breast milk has a potential infectious capacity [69].

Additionally, two studies reported the presence of SARS-CoV-2 IgG in breast milk samples from SARS-CoV-2 positive mothers [45,46]. SARS-CoV-2 IgG was only identified very early after birth (up to 1.5 months later) [46]. Due to the presence of SARS-CoV-2 IgG in two studies, the potential protective role of breast milk against SARS-CoV-2 should be considered [45,46]. The Chinese expert consensus group for managing mothers and neonates with COVID-19 announced that breastfeeding is not recommended [69]. According to the articles reviewed, there is some probability that SARS-CoV-2 infection can be transmitted through breast milk. Due to the SARS-CoV-2 virus being transmitted through respiratory droplets during breastfeeding [4,70], certain recommendations should be taken into consideration if an infected mother decides to breastfeed her neonate. These recommendations have been provided by the Center for Disease Control and Prevention and include wearing a facemask and hand washing before and after breastfeeding [71]. Besides, it has been reported that holder pasteurization, but not freezing, can inactivate the SARS-CoV-2 virus [72].

In summary, in this review, some studies reported that SARS-CoV-2 RNA was found in amniotic fluid, placenta, umbilical cord, and breast milk. Besides, it was found in this review that mothers transmitted SARS-CoV-2 to some neonates through amniotic fluid (*n* = 1 neonate), cervical secretion (*n* = 1), placenta (*n* = 6 neonates), breast milk (*n* = 5 neonates). Findings from this review support the transplacental infection of COVID-19 in certain cases. Nevertheless, it is not yet clear that SARS-CoV-2 can always be transmitted from mother to infant. Future studies assessing the potential transmission of SARS-CoV-2 from mother to infant should clarify this issue. Prospective studies should also address the circumstances that facilitate or prevent SARS-CoV-2 from crossing the placenta barrier.

Limitations of this study include the fact that all the studies included were case reports or case series. An overlapping of participants among studies might be present. Some studies in this review did not perform analyses for amniotic fluid, cord blood, placenta, or breast milk. Future studies should test as many samples as possible in order to find potential sources of transmission of SARS-CoV-2 from mother to child, especially in those cases in which both the mother and the child had a positive test for SARS-CoV-2.

## Figures and Tables

**Table 1:** Detailed information on the studies included in this review.

Reference	City and Country	Study Design	Number of Pregnant Women	Mean Maternal Age (Years)	Number of Fetuses	Trimester of COVID-19 Diagnosis	Maternal Positive Throat Swab for COVID-19	Mean Gestational Age at Birth (Weeks)	Neonatal Positive Throat Swab for COVID-19	Potential Sources of Transmission Studied	Confirmed Source of Transmission	Neonatal Feeding Method	Vaginal Delivery
[47]	Lima (Peru)	Case report	1	41	1	3	1	34	1	Not assessed	None	Formula	0
[48]	New York (USA)	Case series	43	26.9	18	3	43	37	0	Not reported	Not reported	Not reported	10
[11]	Wuhan (China)	Case series	9	29.8	9	3	9	37.2	0	Amniotic fluid, cord blood, and breast milk	None	Not reported	0
[30]	Wuhan (China)	Case series	3	29.7	3	3	3	36.6	0	Placenta	None	Not reported	Not reported
[31]	Wuhan (China)	Case series	5	28.8	5	3	5	39.5	0	Placenta	None	Formula	3
[49]	Wuhan (China)	Case series	4	29	4	3	4	37.1	0	None	None	Formula	1
[50]	Las Palmas Gran Canaria (Spain)	Case report	1	44	1	3	1	29	0	Not reported	Not reported	Not reported	0
[51]	Zanjan (Iran)	Case report	1	27	1	3	1	30	Not reported	Not reported	None	Not reported	1
[23]	Wuhan (China)	Case report	3	29.3	3	3	3	37	0	Umbilical cord blood	None	Not reported	3
[52]	Wuhan (China)	Case-Control	16	30.3	17	3	16	38	0	Not reported	Not reported	Not reported	2
[12]	Wuhan (China)	Case report	1	30	1	3	1	35	0	Serum, urine, feces, amniotic fluid, umbilical cord blood, placenta, and breast milk	None	Not reported	0
[53]	Wuhan (China)	Case series	15	32.5	15	1, 2 and 3	15	37.1	0	Not reported	Not reported	Not reported	1
[54]	Guangdong (China)	Case series	13	29	13	2 and 3	13	32	None	Not reported	None	Not reported	0
[13]	Wuhan (China)	Case series	19	31	19	3	19	36.3	0	Breast milk, amniotic fluid, and cord blood	None	Formula	1
[55]	Chengde (China)	Case report	1	22	1	3	1	38	0	Serum	None	Formula	0
[14]	Wanzhou (China)	Case report	1	25	1	3	1	35.3	0	Amniotic fluid, cord blood, placenta, vaginal secretion, serum, anal sample, breast milk	None	Not reported	0
[15]	Paraná (Brazil)	Case report	1	42	1	2	1	28	0	Placenta, amniotic fluid, umbilical cord blood	Placenta, umbilical cord blood	None	0
[27]	Wuhan (China)	Case report	1	34	1	3	1	40	0	Breast milk	None	Formula	0
[16]	Suzhou (China)	Case report	1	28	1	3	1	30	0	Amniotic fluid, placenta, cord blood	None	Formula	0
[56]	Wuhan (China)	Case series	8	29.8	8	3	8	37.7	Not reported	Not reported	Not reported	Not reported	2
[57]	Wuhan (China)	Case series	23	29	21	1 and 2	19	37	0	Not reported	Not reported	Not reported	2
[17]	Beijing (China)	Case report	1	25	1	3	1	38.4	0	Maternal cervical secretion, maternal rectal, breast milk, amniotic fluid, and placenta	None	Not reported	1
[28]	Wuhan (China)	Case series	13	30.2	13	3	13	38.2	0	Not assessed	None	Not reported	4
[18]	Wuhan (China)	Case series	7	Not reported	7	3	7	36.5	0	Amniotic fluid, cord blood	None	Not reported	0
[24]	Wuhan (China)	Case series	7	32	7	3	7	39	1	Placenta, cord blood	None	Not reported	0
[19]	Wuhan (China)	Case series	2	30.5	2	1	2	Not reported	Not reported	Amniotic fluid	None	Not reported	Not reported
[22]	Tegucigalpa (Honduras)	Case report	1	41	1	3	1	32	0	Not assessed	None	Not reported	0
[20]	Sari (Iran)	Case report	1	22	1	3	1	33	1	Amniotic fluid, cord blood	Amniotic fluid	Formula	0
[58]	Wuhan (China)	Case series	15	30	15	3	15	38	0	Not reported	Not reported	Not reported	0
[59]	Wuhan (China)	Case series	9	27	10	2 and 3	9	35	0	Not assessed	Not assessed	Formula	2
[32]	New York (USA)	Case series	11	31.6	11	3	11	34.4	0	Placenta	Placenta	Not reported	7
[29]	Bergamo (Italy)	Case series	22	Not reported	22	3	22	36	2	Placenta	Placenta	Breastfeeding	0
[25]	Lausanne (Switzerland)	Case report	1	28	1	2	1	19	1	Placenta, cord blood, Maternal cervical secretion	Placenta	None (miscarriage)	0
[33]	Toronto (Canada)	Case report	1	40	1	3	1	35	1	Placenta, breast milk	Placenta, breast milk	Breastfeeding	0
[34]	Boston (USA)	Case series	19	30	19	3	19	38	0	Placenta	Placenta	Not reported	7
[35]	Missouri (USA)	Case report	1	29	1	3	1	40	0	Placenta	Placenta	Breastfeeding	1
[26]	Pune (India)	Case report	1	24	1	3	1	38	1	Umbilical cord stump, placenta	Umbilical cord stump, placenta	Formula	1
[36]	Palermo (Italy)	Case series	15	29	1	3	1	37	1	Placenta	Placenta	Not reported	1
[21]	Paris (France)	Case report	1	23	1	3	1	35	1	Vaginal secretion, placenta, amniotic fluid	Placenta and vaginal secretion	Formula	0
[37]	Connecticut (USA)	Case report	1	35	1	2	1	22	0	Placenta	Placenta	Not provided	0 *
[38]	New York (USA)	Case report	1	40	1	2	1	28	0	Placenta	Placenta	Not provided	0
[39]	Heidenheim (Germany)	Case series	2	Not reported	2	Not reported	2	Not reported	1	Breast milk	Breast milk	Breastfeeding	Not reported
[40]	Adelaide (Australia)	Case report	1	40	1	Postpartum	1	8 months (postpartum)	1	Breast milk	Breast milk	Breastfeeding	Not reported
[41]	Ankara (Turkey)	Case report	1	20	1	3	1	38	1	Breast milk	Breast milk	Breastfeeding	1
[45]	Zhejiang (China)	Case report	1	32	1	Postpartum	1	13 months (postpartum)	1	Breast milk, feces, maternal serum	Breast milk, maternal serum	Breastfeeding	Not reported
[42]	Modena (Italy)	Case report	1	33	1	3	1	32	1	Breast milk	Breast milk	Breastfeeding	0
[43]	Los Angeles (USA)	Case series	18	34.4	18	3 and postpartum	18	Not reported	Not reported	Breast milk	Breast milk	Not reported	Not reported
[46]	Shangai (China)	Case report	1	33	1	3	1	38	0	Vaginal secretion, maternal serum, breast milk	None	Not reported	1
[44]	Wuhan (China)	Case series	5	33	5	Postpartum	5	36	0	Vaginal secretion, serum, breast milk	Breast milk	Not reported	1

* Dilation and evacuation termination of delivery.
